# Non-destructive real-time analysis of plant metabolite accumulation in radish microgreens under different LED light recipes

**DOI:** 10.3389/fpls.2023.1289208

**Published:** 2024-01-11

**Authors:** Marco Garegnani, Carla Sandri, Claudia Pacelli, Francesca Ferranti, Elisabetta Bennici, Angiola Desiderio, Luca Nardi, Maria Elena Villani

**Affiliations:** ^1^ ENEA, Italian National Agency for New Technologies, Energy and Sustainable Economic Development, Department for Sustainability Casaccia Research Center, Rome, Italy; ^2^ Department of Aerospace Science and Technology, Politecnico of Milano, Milan, Italy; ^3^ Human Spaceflight and Scientific Research Unit, Italian Space Agency, Rome, Italy

**Keywords:** microgreen, hydroponic, space environment, LED lights, fluorescence-based non-destructive techniques

## Abstract

**Introduction:**

The future of human space missions relies on the ability to provide adequate food resources for astronauts and also to reduce stress due to the environment (microgravity and cosmic radiation). In this context, microgreens have been proposed for the astronaut diet because of their fast-growing time and their high levels of bioactive compounds and nutrients (vitamins, antioxidants, minerals, etc.), which are even higher than mature plants, and are usually consumed as ready-to-eat vegetables.

**Methods:**

Our study aimed to identify the best light recipe for the soilless cultivation of two cultivars of radish microgreens (Raphanus sativus, green daikon, and rioja improved) harvested eight days after sowing that could be used for space farming. The effects on plant metabolism of three different light emitting diodes (LED) light recipes (L1—20% red, 20% green, 60% blue; L2—40% red, 20% green, 40% blue; L3—60% red, 20% green, 20% blue) were tested on radish microgreens hydroponically grown. A fluorimetric-based technique was used for a real-time non-destructive screening to characterize plant methabolism. The adopted sensors allowed us to quantitatively estimate the fluorescence of flavonols, anthocyanins, and chlorophyll via specific indices verified by standardized spectrophotometric methods. To assess plant growth, morphometric parameters (fresh and dry weight, cotyledon area and weight, hypocotyl length) were analyzed.

**Results:**

We observed a statistically significant positive effect on biomass accumulation and productivity for both cultivars grown under the same light recipe (40% blue, 20% green, 40% red). We further investigated how the addition of UV and/or far-red LED lights could have a positive effect on plant metabolite accumulation (anthocyanins and flavonols).

**Discussion:**

These results can help design plant-based bioregenerative life-support systems for long-duration human space exploration, by integrating fluorescence-based non-destructive techniques to monitor the accumulation of metabolites with nutraceutical properties in soilless cultivated microgreens.

## 1 Introduction

International space agencies and private industries are entering a new phase of sustainable human exploration of space beyond low Earth orbit. One of the key challenges is to recreate conditions suitable for human survival and well-being, providing water, food (Anderson et al., 2018), air, and shelter, reducing the amount of re-supply required from Earth. This issue has been addressed by developing several types of life support systems (LSS) over the years, carrying out numerous experiments in space and for analog missions on Earth (Bamsey et al., 2015). The introduction of biological processes to regenerate resources is a reasonable enhancement to LSS, and it is the first step toward demonstrating the concept of complex biological life support systems (BLSS), which, when combined with other physiochemical technologies, could improve resource recycling.

The most important aspects to be considered are the adequate energy intake, quality, and nutritive value of the food consumed by astronauts. Exploration missions to Mars may require nutritional quality stability for up to five years (Douglas et al., 2021). During this time, the space environment, radiation, microgravity, isolation, confinement, and distance from Earth have a powerful impact on both physiological and psychological health (Landon et al., 2019). The production of fresh food could serve as an important countermeasure to these deleterious effects (Douglas et al., 2020). To test the effects of the space environment on plants, experimental research is often conducted in dedicated environmentally controlled cultivation chambers (Monje et al., 2003) controlling air temperature, relative humidity, water and nutrients, light quality, and intensity. Controlled closed environmental systems allow you to evaluate and characterize the effects of single or multiple stressors by analyzing plant responses using imaging technologies that can reveal stresses at early stages (Zhao et al., 2019; Yang et al., 2020). Moreover, using such imaging technologies, it is possible to collect data for quantitative studies of complex traits related to growth, yield, and adaptation to biotic and abiotic stress. They are recognized as the only tool able to deliver an accurate description of trait expression in multiple-stress environments (Li et al., 2014; Zhao et al., 2019). Visible light imaging provides information on the canopy cover and color, and it is the simplest technology for monitoring plant architecture attributes such as leaf/cotyledon area, color, growth dynamics, seedling vigor, seed morphology, and image-based projected biomass (Li et al., 2014). Fluorescence imaging provides a rapid real-time non-invasive screening technique to identify plants with altered metabolism and growth using specific fluorometric devices (Multiplex and Dualex, A-Force France). This can monitor different physiological processes and estimate, in real-time, accumulated compounds such as flavonols, anthocyanins, and chlorophyll (Flav, Anth, Chl) (Sytar et al., 2015; Zivcak et al., 2017; Brazaitytė et al., 2019). The specific red (RF) and far‐red (FRF) fluorescence signals from Flav, Anth, and Chl are obtained from the leaves/cotyledons at various excitation bands (UV-A 370 nm, blue 460 nm, green 516 nm, and red 637 nm).

In the space farming context, microgreens have recently gained popularity as ready-to-eat, healthy, and fast-growing food (Xiao et al., 2012; Kyriacou et al., 2016; Kyriacou et al., 2017; Sharma et al., 2022). Microgreens consist of the seedlings harvested one or two weeks after germination, at the appearance of the first true leaves. Compared to mature-leaf crops, they contain from 4 to 40 times higher concentrations of bioactive compounds such as vitamins, antioxidants, minerals, and phytonutrients (Kyriacou et al., 2019; Bhaswant et al., 2023). Their consumption, thanks to the high nutritional values, antioxidant, anti-inflammatory, and anti-diabetic effects, attenuates chronic diseases and typical effects of the space environment on the human body. Moreover, it reduces the risk of cancer, respiratory problems, osteoporosis, and muscle atrophy (Alekel et al., 2000; Tada and Yokogoshi, 2002; Choe et al., 2018; Teng et al., 2021). Microgreens have delicate textures, and intense and distinctive flavors (Renna et al., 2017), thanks to their organoleptic traits that are nowadays widely used in high-quality restaurants as salads or garnishes. Since their taste is species-dependent, flavors can range from high bitterness to moderate and spicy (Sharma et al., 2022); providing a large assortment of options to address astronaut menu fatigue (Tang et al., 2022). Many leafy greens, herbs, and root crops can becultivated as microgreens, but the most used come from the Brassicaceae and Amaranthaceae families (Kyriacou et al., 2016; Xiao et al., 2016). Radish microgreens, a member of the Brassicaceae family, are an ideal candidate for space cultivation due to their fast growth rates, high yields, ease of production, and spicy flavor (Kyriacou et al., 2017). Red-purple varieties are rich in anthocyanins, which are strong antioxidant molecules able to counteract the oxidative damage caused by chronic exposure to cosmic radiations and the detrimental effects of living in extreme and confined environments under very stressful conditions such as long-term space missions (Mattioli et al., 2020; Alam et al., 2021).

Light plays a crucial role in microgreen cultivation, and it is one of the most important environmental factors that affects plant development and physiology (Kyriacou et al., 2016).

Several studies have been conducted on microgreens, which highlighted that the activation of specific metabolic pathways is light-dependent (Alrifai et al., 2019; Zhang et al., 2020).

For space farming applications, light must be provided efficiently to plants because it draws a significant portion of the total power consumption of the cultivation system. Therefore, careful optimization is fundamental to reduce resource utilization and maximize plant production, both in terms of biomass and metabolite content. Despite variations in efficiency among LEDs of different wavelengths (Kusuma et al., 2020), optimizing the light spectrum, intensity, and photoperiod can result in improved yields and quality.

Different red-to-blue (R:B) ratios affect microgreen growth and development, influencing parameters such as leaf area and fresh weight, biomass accumulation, stem elongation, chlorophyll, flavonol, anthocyanin, and carotenoid concentration. Generally, the changes in R:B ratio trigger specific responses in different genotypes; however, several studies agree that a higher percentage of blue light affects preferentially plant secondary metabolite concentration in brassica microgreens (Kopsell et al., 2014; Vaštakaitė et al., 2015; Samuolienė et al., 2017; Brazaitytė et al., 2021).

The same effect was observed with blue light for the accumulation of macro- and micro-nutrients in mustard and kale microgreens. Bantis (2021) studied three different R:B (with their peaks at 600 nm and 650 nm for red, at 450 nm for blue) ratios: 2:1, 5:1, and 9:1. A higher blue percentage induced in mustard microgreens a higher total carotenoid content, and a 5:1 ratio induced higher total carotenoid content in radish and peas shoots. A lower blue percentage (9:1) induced higher biomass in basil and mustard while in radish caused higher total phenolic content. Different ratios had no effect on biomass accumulation in radish plants. In mustard microgreens, the combined treatment of red and blue light (450 nm + 650 nm) promoted the accumulation of total anthocyanin, while the blue (450 nm) light was found to be the dominant factor in the accumulation of non-anthocyanin phenolics (Liu et al., 2022).

The inclusion of green wavelengths (500–600 nm) to red and blue light promotes the growth (dry weight biomass) (Kim et al., 2004; Mickens et al., 2018) and the accumulation of bioactive phytochemicals in different species (Orlando et al., 2022). Microgreens grown under green light displayed higher nutrient content compared to those grown under red and blue light only (Gerovac et al., 2016). In addition, mineral accumulation was shown to be stimulated by the inclusion of green light in the red and blue recipe. For example, mustard microgreens accumulated 26%, 13%, and 10% more K, Ca, and B, respectively, when grown under a light ratio of R74:G18:B8 compared with those grown under R84:FR7:B9 (Gerovac et al., 2016). Broccoli microgreens responded with higher nutrient concentration and better yield under RGB light compared to different ratios of red and blue (Kopsell et al., 2014).

Many studies in the literature showed that the integration of far-red (700–750 nm) and UV-A (315–400 nm) wavelengths stimulate secondary metabolite accumulation (Brazaityte et al., 2015; Brazaityte et al., 2019; He et al., 2021). In some species, far-red light induced an increase in biomass production when compared to lights with no far-red. For example, in radish, fresh and dry weight was increased by 25% after the addition of far-red to the RB recipe (Demir et al., 2023). In general, far-red radiation induced stronger effects on microgreens than UV-A when added to a white full-spectrum light (Hooks et al., 2022). The effects of UV induction of secondary metabolism accumulation are highly dependent on the wavelength selected (UV A-B-C) and the genetic background. In fact, many of the effects on growth and development exerted by UV-A are distinct from those triggered by UV-B and vary considerably in terms of the direction the response takes (Verdaguer et al., 2017). However, the introduction of UV radiation benefits the phytochemicals accumulation in plants (Goto et al., 2016; Artés-Hernández et al., 2022). For example, in two varieties of basil microgreens, the supplemental UV-A lighting enhanced the antioxidant properties of green-leaf basil. Purple-leaf basils were shown to be more sensitive to UV-A irradiation and exhibited fewer positive effects on antioxidant properties (Vaštakaitė et al., 2015). In beet microgreens, UV-A radiation at an intensity of 12 μmol m^-2^s^-1^ induced the accumulation of phytochemicals, such as vitamin E, and in general increased antioxidant properties (Sakalauskienė et al., 2014).

Under controlled conditions and with the appropriate combination of image analysis technologies and methods (Chaerle et al., 2007; Zhao et al., 2019), it is possible to monitor physiological processes and provide acceptable phenotyping results (Li et al., 2014). The interpretation of sensor-driven data, essential for the detection of plant stress and optimal harvest timing, requires the use of advanced methods of data acquisition and image analysis. Specialized software and tools, as discussed by the work of Lobet et al. (2013), are indispensable to identifying physical and biochemical alterations connected with the experimental condition tested (Bauckhage and Kersting, 2013; Singh et al., 2016).

In this study, we exploited an integrated approach to evaluate the influence of various light recipes on the growth and metabolic responses of two radish microgreen cultivars. This analysis combines data derived from visible image-based morphometric measurements, biomass quantification, and fluorescence-based indices obtained through the Multiplex instrument. The innovative approach adopted has been utilized to evaluate the feasibility of using fluorescence sensing as a non-invasive method for determining crop status and quality. The results obtained will help in developing and customizing a future modular system integrated with image-based and fluorescence sensors. It will be particularly suited for the space environment where there is a need for highly reliable, real-time, non-destructive methods and techniques for the analysis of ready-to-eat food products to support the diet of astronauts.

## 2 Materials and methods

### 2.1 Plant material

The microgreen species selected were green daikon radish (*Raphanus sativus* L. var. longipinnatus, Italian Sprout Srl, Cesena, Italy) and rioja improved radish (*Raphanus sativus* L., CN seeds Ltd, England).

### 2.2 Seed sowing and growth conditions

Seeds were weighted in a pool of 400 seeds for each technical replica, sterilized with a 2% hydrogen peroxide solution for 10 minutes in dedicated independent plastic containers, then rinsed twice and soaked in aerated distilled water for 24 hours in darkness.

**Figure 1:**
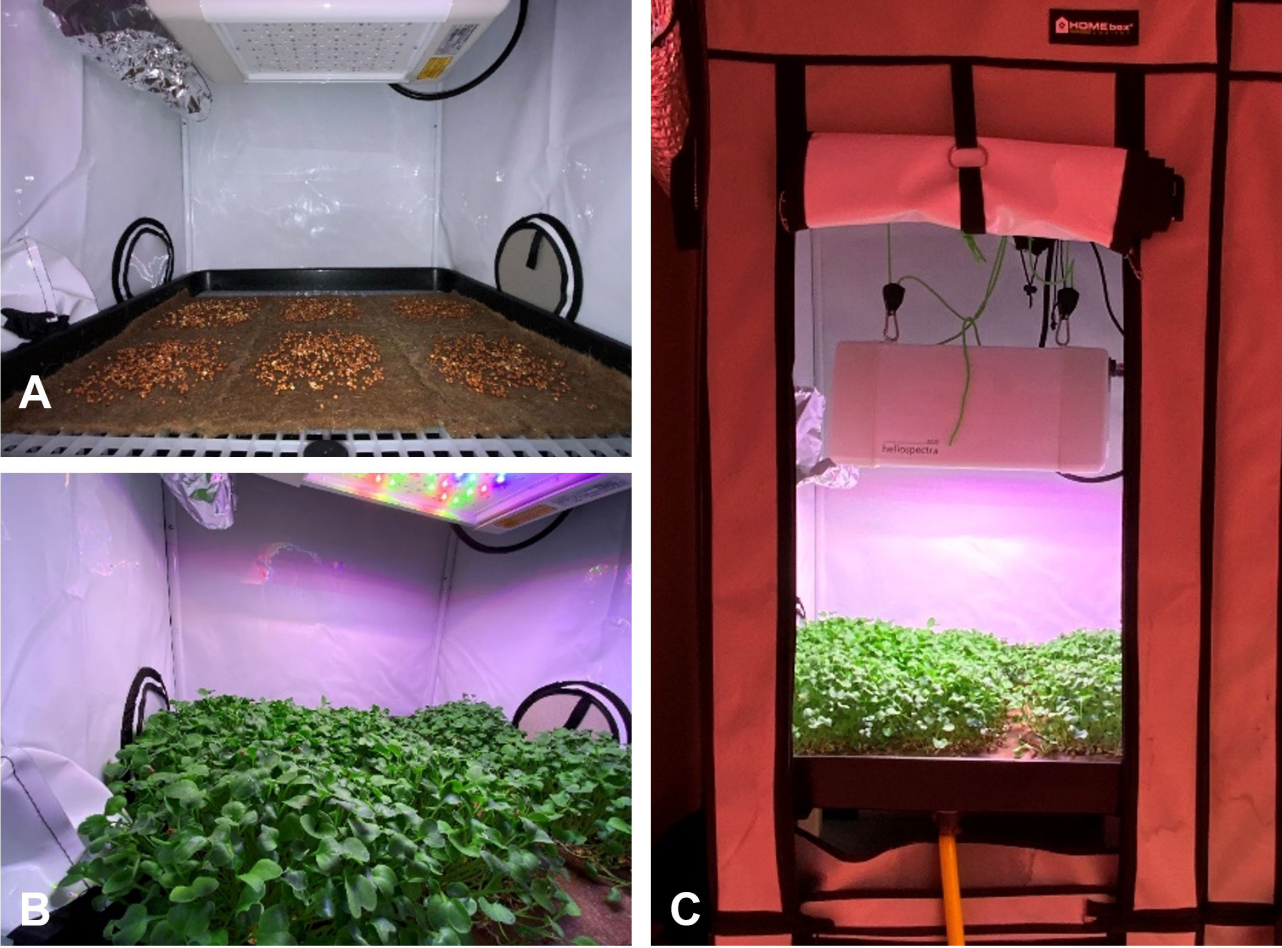
Microgreens cultivation layout: **(A)** Greenfelt pads after sowing with green daikon radish; **(B)** green daikon radish at the harvest time, at 8 DAS; **(C)** HOMEbox (Germany) grow-room before harvest.

After this treatment, seeds were sowed onto five pads (20×20×0.5 cm) of an organic substrate (Greenfelt, Manifattura Maiano, Firenze, Italy), a fully biodegradable felt containing a mix of recycled jute (85%) and kenaf (15%). Each pad was sowed at a density of 1 seed/cm^2^, with a total of 400 seeds per pad. Radish microgreens were cultivated in an “ebb and drain” hydroponic system, seeds were evenly distributed on the substrate, and the nutrient solution (Idrofill Base NPK 10-5-23 (8Ca-2Mg) 1 g/l, K-Adriatica, Rovigo, Italy) diluted in distilled water was pumped into each tray (Garland Ebb & Flood, 60x60x6 cm, West Midlands, England) and then drained back into the reservoir. This process was repeated every two hours. The grow box (HOMEbox Ambient Q60+, 60x60x160 cm, Berlin, Germany) was installed inside a sterile growth chamber (ISO 5 class clean room) and equipped with a TURBO 100 ventilator (10 cm diameter, 187 m^3^/h, Blauberg, Germany), as shown in **Figure 1**. During the experiment, daily air temperature in the growth chamber was set at 22 ± 1°C and relative humidity at 60%. After sowing, seeds were germinated three days in darkness, and microgreens were harvested eight days after sowing (DAS).

### 2.3 Light recipes

**Figure 2:**
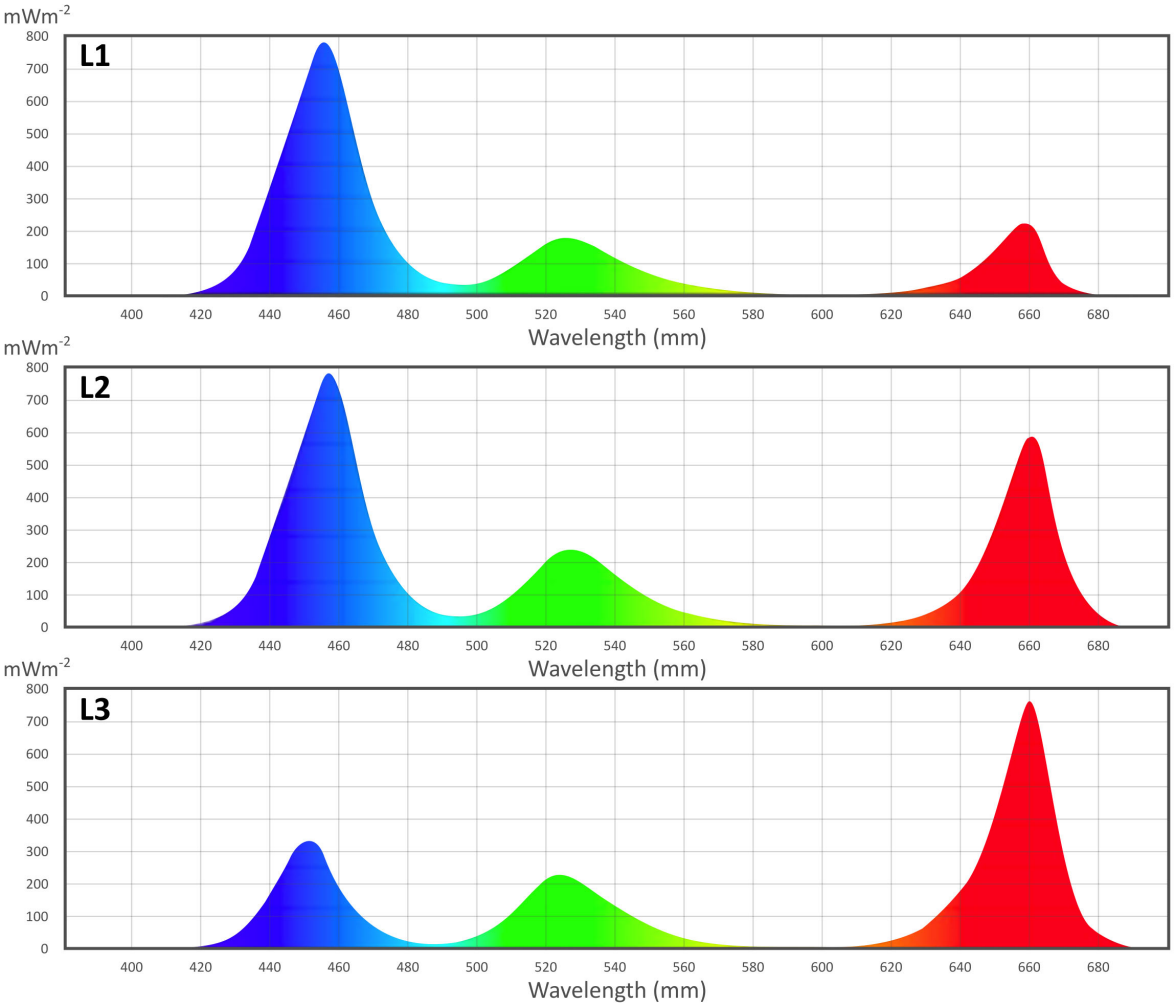
First trial light recipe spectra, measured with MK350S Premium spectroradiometer (Uptrek, Taiwan).

All LED light treatments were provided by RX30 “DYNA” LED lamps (Heliospectra, Goteborg, Sweden), with nine spectral channels (380, 400, 420, 450, 520, 630, 660, and 735 nm, and 5700K white) individually regulated. Lamps were positioned at 50 cm from the cultivation area. In the first trial, a combination of three single wavelengths of LED light recipes was used: blue light at 450 nm, red at 660 nm, and green at 520 nm. Three recipes (as shown in **Figure 2**) were defined as L1 (20% red, 20% green, 60% blue), L2 (40% red, 20% green, 40% blue), and L3 (60% red, 20% green, 20% blue). The total intensity was set at 150 μmol m^-2^s^-1^ for each light recipe. In the second trial, 10 μmol m^-2^s^-1^ UV-A (380 nm) and/or far-red (735 nm) lights were integrated into the best-performing LED light recipe (L∗) obtained from the first trial. Light measurements (intensity and spectra) were performed with a spectroradiometer (MK350S Premium spectroradiometer UPRtek, Taiwan) at the cultivation tray level. The photoperiod was set at 12 hours light time and 12 hours darkness, resulting in higher energy use efficiency and 25% lower power consumption than the traditional photoperiod of 16 hours light time and 8 hours darkness (Hernández-Adasme et al., 2023).

### 2.4 Plant measurements

**Figure 3:**
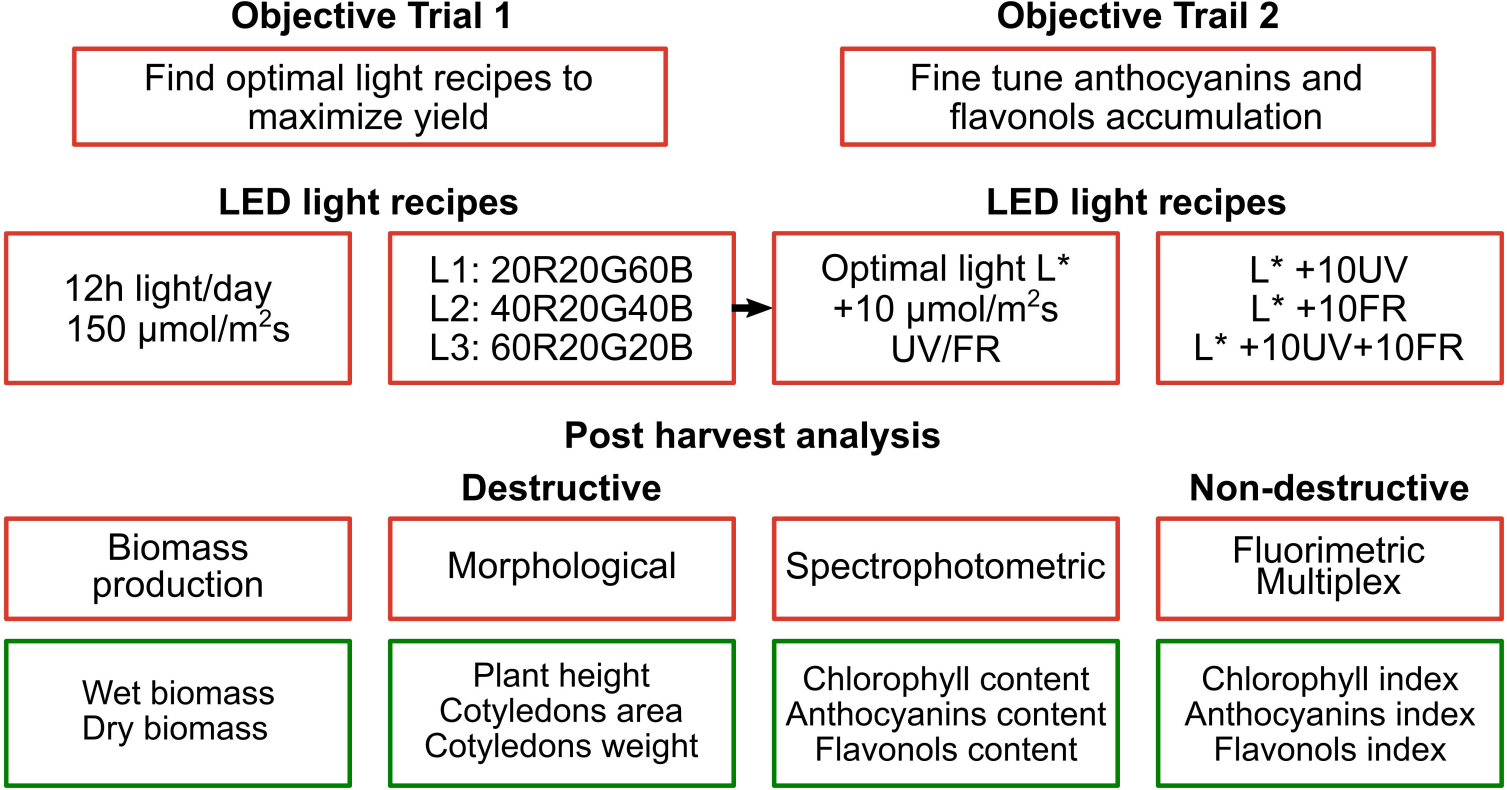
Graphical representation of the experimental design. Note that the optimal light selected in the first trial L* is integrated with UV and/or far-red light in the second trial.

Radish microgreens were harvested at 8 DAS, and three of the five seeded pads were selected for the analysis. In each pad, representing a technical replica, 20 seedlings were harvested, and their morphometrical parameters (total fresh and dry weight, cotyledon weight and area, and hypocotyl length) were measured. To measure fresh weight, a precision analytical balance (AS X2 RadWag, Poland) was used, and for the dry weight a moisture analyzer (MA.R Moisture Analyzer Radwag, Poland). The hypocotyl length was measured with a digital caliper (Digi-MaxTM slide caliper, Sigma-Aldrich). Leaf area was analyzed using ImageJ software (Java-based digital image processing computer program), taking a photo of the seedling perpendicular to the horizontal surface on a white background with a reference dimension (1 cm segment). The complete experimental plan, as previously discussed, is reported in **Figure 3**.

### 2.5 Statistical analysis

A one-way analysis of variance (ANOVA) with multiple comparisons Tukey test was conducted to determine if there were significant differences between the means of the test groups. P values are reported: ns (P>0.05); * (P ≤ 0.05); ** (P ≤ 0.01); *** (P ≤ 0.001); and **** (P ≤ 0.0001). The analyses were performed using GraphPad Prism 8.4 software (Boston, USA).

### 2.6 Non-destructive metabolite analysis

The adaxial and abaxial sides of the radish cotyledons were separately analyzed with a handheld multi-parametric fluorescence sensor (Multiplex 3 Force-A, Orsay, France), based on light-emitting diode excitation and filtered-photodiode detection (Ghozlen et al., 2010). The sensor illuminates a circular surface of 8 cm in diameter at a distance of 10 cm from the source and provides 12 signals and several signal ratios. For this study, we used the chlorophyll-related SFR index, anthocyanins-related ANTH, and flavonols-related FLAV indexes sum of adaxial and abaxial values as described by Diago et al. (2016).

### 2.7 Metabolite extraction and analysis

Once radish microgreens were harvested, nine pools (three samples per metabolite) of seven seedlings for each pad (three replicas for each light condition) were sampled, frozen in liquid nitrogen, and then stored at –80°C for further analysis.

#### 2.7.1 Chlorophyll extraction

Each sample was homogenized with a homogenizer (Ultraturrax T8 mm tool for 30 sec at 5000 rpm) adding 2 ml of 80% acetone in distilled Milli-Q water, then centrifuged for 10 minutes at 15000 rpm, and the supernatant was analyzed by spectrophotometer (Amersham, Pharmacia). The supernatant was read at four different wavelengths: 470 nm, 646 nm, 663 nm, and 720 nm. Pigment content was computed according to the following equations (Harmut, 1987):


$$\text{C}{\text{H}}_{\text{A}}=\;12.25\;{\text{A}}_{663}–\;2.79\;{\text{A}}_{646}\text{ C}{\text{H}}_{\text{B}}=\;21.50\;{\text{A}}_{646}–\;5.10\;{\text{A}}_{663}\text{ C}{\text{H}}_{\text{Tot}}{=}_{\text{A}}+\text{C}{\text{H}}_{\text{B}}$$

#### 2.7.2 Anthocyanin extraction

Each sample was homogenized with a homogenizer (Ultraturrax T8 mm tool for 30 sec at 5000 rpm), in 2 ml methanol with 50 mM of chloridric acid and incubated overnight at –20°C. The day after, 2 ml of chloroform and 1.5 ml of distilled Milli-Q water were added, and samples were centrifuged for 5 minutes at 15000 rpm. The upper phase was taken and read at 535 nm. Concentration (expressed in ng/µl) was computed as linear regression from the measurement of known concentrations of delphidin glucoside.

#### 2.7.3 Flavonol extraction

Each sample was homogenized with a homogenizer (Ultraturrax T8 mm tool for 30 sec at 5000 rpm) in 2 ml of 75% methanol and 0.1% formic acid in distilled water, vortexed, and shaken for 10 min at RT. Then samples were kept steady at room temperature for 15 minutes and shaken again for 10 min. Then samples were centrifuged for 15 minutes at 15000 rpm, and the supernatant was analyzed at 425 nm. Concentration (expressed in ng/ul) was computed as linear regression from the measurement of known concentrations of quercetin.

## 3 Results

### 3.1 Effects of LED lights on radish microgreens’ growth and development

**Figure 4:**
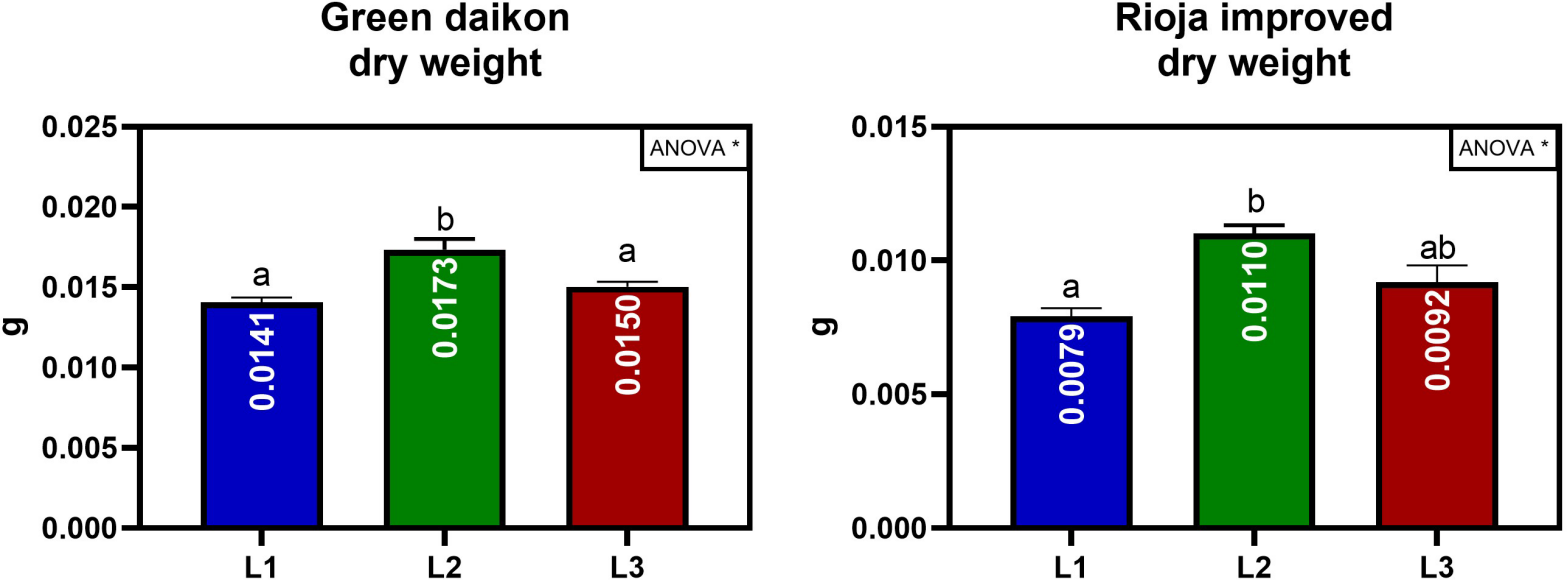
Total dry weight per seedling of green daikon and rioja improved radish microgreens, grown under L1, L2, and L3 lights. Error bars show the standard error of the mean (SEM). In the graph, the results of a one-way ANOVA analysis are reported. P value: ∗ (P ≤ 0.05). Different letters mean significant results with P<0.05.

The effect on biomass accumulation and morphological parameters of the three LED light spectra (L1, L2, and L3) were studied on two microgreens: green daikon and rioja improved radish. The results of green daikon radish grown under the L1, L2, and L3 light recipes are reported in **Table 1**. The effect of L1 light resulted in a statistically significant decrease in total fresh weight (cotyledons + hypocotyl) concerning L2 and L3. The cotyledonal area showed a similar trend: L2 light induced a statistically significant increase compared to L1 and L3. No statistically significant effects were observed on hypocotyl length between treatments, although plants grown under L3 presented longer stems. The effect of L2 light on total dry weight was statistically significant compared to L1 and L3, resulting in higher biomass accumulation, as shown in **Figure 4**.

**Table 1:** Morphological parameters and chlorophyll content of green daikon radish microgreens grown under L1 (20% red, 20% green, 60% blue), L2 (40% red, 20% green, 40% blue), and L3 (60% red, 20% green, 20% blue) lights.

Parameter	L1	L2	L3
Fresh weight (g)	0.262 ± 0.060 a	0.353 ± 0.061 b	0.323 ± 0.048 b
Fresh cotyledon weight (g)	0.138 ± 0.102 a	0.183 ± 0.041 b	0.151 ± 0.028 a
Hypocotyl length (mm)	57.6 ± 10.9 a	68.3 ± 9.5 a	74.4 ± 10.9 a
Cotyledonal area (mm^2^)	419 ± 116 a	584 ± 142 b	471 ± 96 a
Dry weight (g)	0.014 ± 0.003 a	0.017 ± 0.005 b	0.015 ± 0.003 a
Chlorophyll concentration (mg/gFW)	0.842 ± 0.205 a	0.494 ± 0.116 b	0.552 ± 0.083 b

Data reported are mean ± standard deviation. Different letters mean significant results with P<0.05.

The results of rioja improved radish cultivation under the treatment with L1, L2, and L3 lights are reported in **Table 2**. The effect of L1 light resulted in a statistically significant decrease in total fresh weight (cotyledons + hypocotyl) concerning L2 and L3. The effect of L2 on cotyledonal fresh weight was statistically significant, showing larger cotyledons than L1 and L3 light. No statistically significant effects were observed on the cotyledonal area; however, L2 presented slightly larger cotyledons. The effect on hypocotyl length showed different results: L3 significantly stimulated plant elongation compared to L1. The effect of L2 light on dry weight was statistically significant compared to L1, resulting in higher biomass accumulation, as shown in **Figure 4**.

**Table 2:** Morphological parameters and chlorophyll content of rioja improved radish microgreens grown under L1 (20% red, 20% green, 60% blue), L2 (40% red, 20% green, 40% blue), L3 (60% red, 20% green, 20% blue) lights.

Parameter	L1	L2	L3
Fresh weight (g)	0.146 ± 0.028 a	0.212 ± 0.050 b	0.187 ± 0.111 b
Fresh cotyledon weight (g)	0.072 ± 0.018 a	0.110 ± 0.027 b	0.070 ± 0.022 a
Hypocotyl length (mm)	48.4 ± 7.6 a	54.2 ± 12.2 ab	61.6 ± 10.9 b
Cotyledonal area (mm^2^)	221 ± 47 a	244 ± 130 a	215 ± 59 a
Dry weight (g)	0.008 ± 0.002 a	0.011 ± 0.003 b	0.009 ± 0.005 ab
Chlorophyll concentration (mg/gFW)	0.564 ± 0.111 a	0.377 ± 0.071 b	0.562 ± 0.083 a

Data reported are mean ± standard deviation. Different letters mean significant results with P<0.05.

### 3.2 Effects of UV and far-red on radish microgreens growth and metabolite accumulation

**Figure 5:**
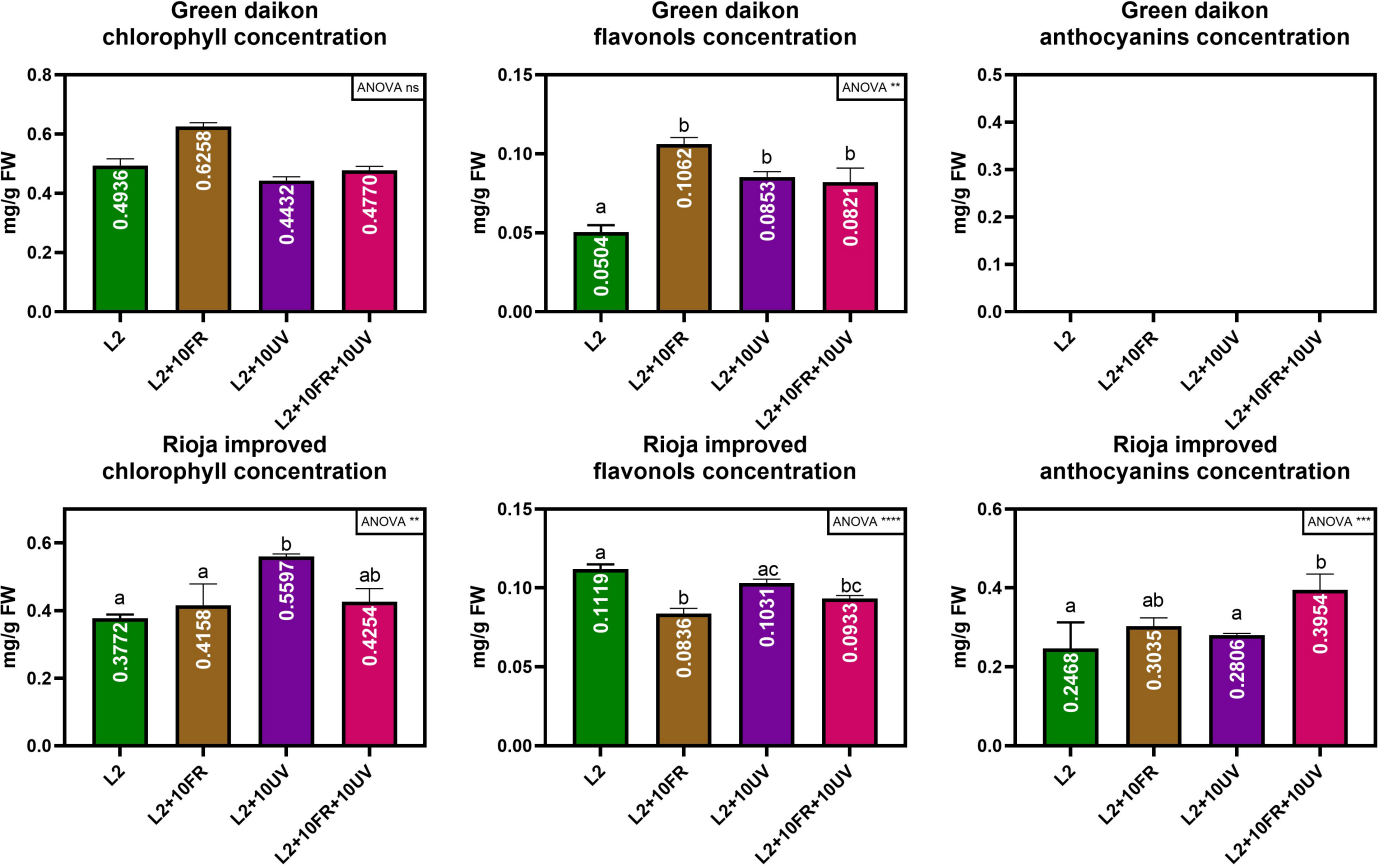
Chlorophyll, anthocyanin, and flavonol concentration of green daikon and rioja improved radish microgreens, grown under +10UV, +10FR, and +10UV+10FR, with L2 reported as control. Error bars show the standard error of the mean (SEM). In the graph, the results of a one-way ANOVA analysis are reported. P value: ns (P>0.05); ∗∗ (P ≤ 0.01); ∗∗∗ (P ≤ 0.001); ∗∗∗∗ (P ≤ 0.0001). Different letters mean significant results with P<0.05.

The second trial focused on fine-tuning the best light recipe previously found (L2) to increase microgreens’ nutritional quality. UV-A and far-red lights were integrated into the L2 recipe, because of their effects on secondary metabolite accumulation. This trial tested three different variations of the L2 recipe: L2 + UV-A, L2 + far-red, and L2 + UV-A and far-red. The results on the morphological parameters and metabolite concentrations of green daikon radish under the treatment with L2 + 10UV, L2 + 10FR, or L2 + 10UV+10FR lights are reported in **Table 3**. The effects of UV and far-red alone were statistically significant for fresh and dry weight, and cotyledon area and weight: plants grown under these conditions showed lower biomass accumulation and smaller cotyledons. The hypocotyl length showed a different trend: only the combination of UV and far-red light had a statistically significant effect on hypocotyl elongation. Metabolite concentrations are reported in **Figure 5**, where the concentration values of anthocyanins for the green cultivar have been omitted because they exhibited concentrations below the sensitivity threshold of the spectrophotometer. The different light recipes did not have a statistically significant effect on chlorophyll concentration in the cotyledons; however, only far-red light induced a slightly higher concentration than other recipes. The integrated lights had a statistically significant effect on the accumulation of flavonols inside cotyledons compared to L2.The results of rioja improved radish cultivation grown under the treatment with L2 + 10UV, L2 + 10FR, or L2 + 10UV+10FR lights are reported in **Table 4**. The effect of UV and far-red combination was statistically significant on fresh weight and hypocotyl length: plants grown under these conditions showed higher fresh biomass and elongated stem compared to L2 and L2 + 10UV lights. Dry weight, cotyledon area, and weight showed no statistically significant differences when treated under different light treatments. However, UV alone induced a slight decrease in dry weight and cotyledons’ fresh weight. Chlorophyll, anthocyanins, and flavonol concentrations are reported in **Figure 5**. Exposure to UV light alone induced a statistically significant increase in cotyledon chlorophyll concentration compared to other lights. The L2+UV+FR light effect on anthocyanin concentration was statistically significant. Plants grown under these conditions showed an increase in anthocyanin accumulation. The integration of far-red and both far-red and UV lights induced a statistically significant decrease in flavonol concentration compared to L2 light.

**Table 3:** Morphological parameters and metabolites content of green daikon radish microgreens grown under L2, L2 + 10UV, L2 + 10FR, and L2 + 10UV+10FR lights.

Parameter	L2	L2 + 10UV	L2 + 10FR	L2 + 10UV+10FR
Fresh weight (g)	0,353 ± 0.061 a	0.214 ± 0.033 b	0.253 ± 0.050 b	0.331 ± 0.055 a
Fresh cotyledon weight (g)	0.183 ± 0.041 a	0.099 ± 0.020 b	0.114 ± 0.027 b	0.153 ± 0.037 a
Hypocotyl length (mm)	68.3 ± 9.5 a	61.1 ± 7.4 a	66.7 ± 9.7 a	79.8 ± 8.9 b
Cotyledonal area (mm^2^)	584 ± 142 a	282 ± 69 b	332 ± 89 b	441 ± 117 a
Dry weight (g)	0.0174 ± 0.005 a	0.013 ± 0.003 b	0.014 ± 0.002 b	0.016 ± 0.003 ab
Chlorophyll concentration (mg/gFW)	0.494 ± 0.116 a	0.443 ± 0.160 a	0.626 ± 0.063 a	0.477 ± 0.135 a
Flavonol concentration (mg/gFW)	0.0504 ± 0.125 a	0.085 ± 0.011 b	0.106 ± 0.014 b	0.082 ± 0.024 b

Data reported are mean ± standard deviation. Different letters mean significant results with P<0.05.

**Table 4:** Morphological parameters and metabolites content of rioja improved radish microgreens grown under L2, L2 + 10UV, L2 + 10FR, and L2 + 10UV+10FR lights.

Parameter	L2	L2 + 10UV	L2 + 10FR	L2 + 10UV+10FR
Fresh weight (g)	0.212 ± 0.050 ab	0.191 ± 0.043 a	0.239 ± 0.055 bc	0.253 ± 0.055 c
Fresh cotyledon weight (g)	0.110 ± 0.027 a	0.0974 ± 0.032 a	0.109 ± 0.032 a	0.105 ± 0.031 a
Hypocotyl length (mm)	54.2 ± 12.2 ab	52.1 ± 9.9 a	62.6 ± 10.8 bc	71.2 ± 8.4 c
Cotyledonal area (mm^2^)	245 ± 130 a	238 ± 46 a	240 ± 53 a	239 ± 59 a
Dry weight (g)	0.011 ± 0.003 a	0.010 ± 0.002 a	0.011 ± 0.002 a	0.012 ± 0.004 a
Chlorophyll concentration (mg/gFW)	0.377 ± 0.071 a	0.560 ± 0.041 b	0.416 ± 0.137 a	0.425 ± 0.138 ab
Flavonol concentration (mg/gFW)	0.112 ± 0.008 a	0.103 ± 0.008 ac	0.084 ± 0.009 b	0.093 ± 0.007 bc
Anthocyanin concentration (mg/gFW)	0.247 ± 0.114 a	0.281 ± 0.037 b	0.304 ± 0.073 a	0.395 ± 0.113 b

Data reported are mean ± standard deviation. Different letters mean significant results with P<0.05.

### 3.3 Non-destructive diagnostic techniques analysis

**Figure 6:**
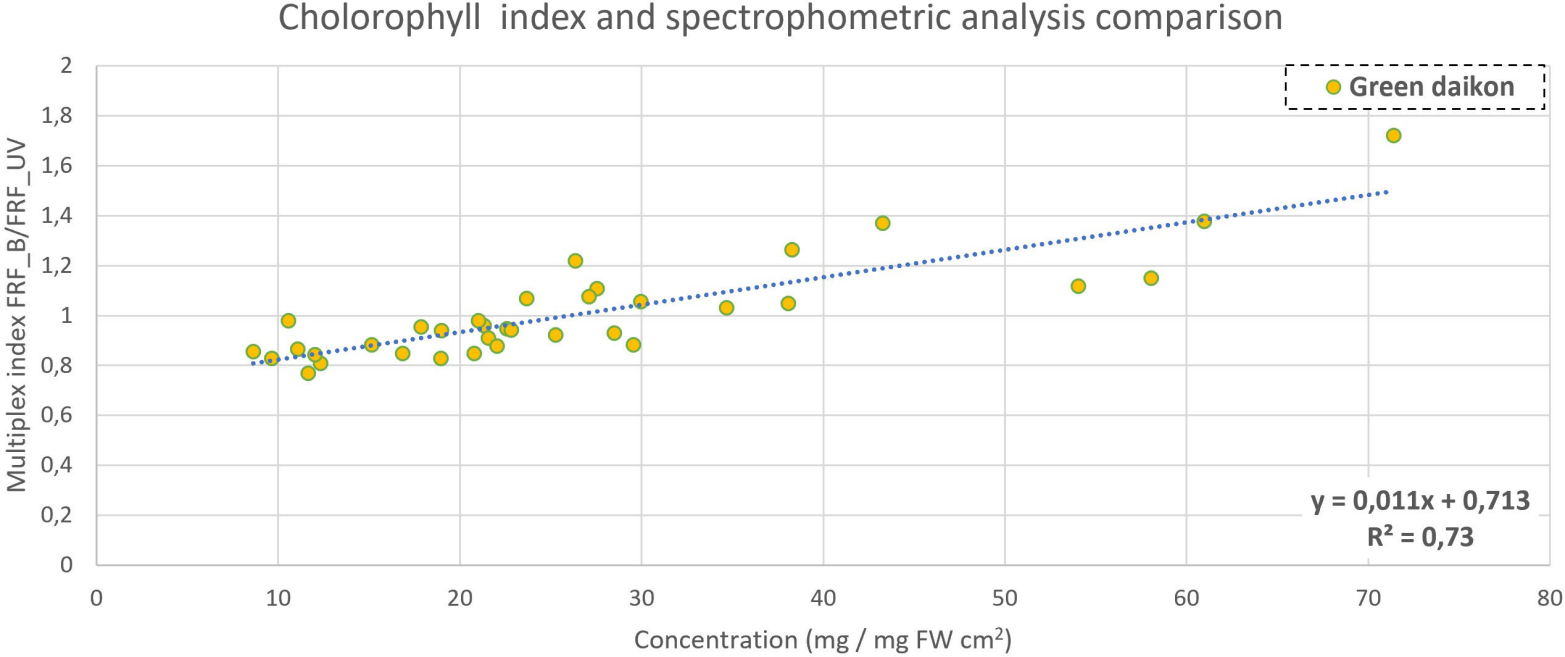
Chlorophyll content comparison between Multiplex index SFR (measuring adaxial side of cotyledon) and concentration in green daikon radish, with a P-value<0.001.

**Figure 7:**
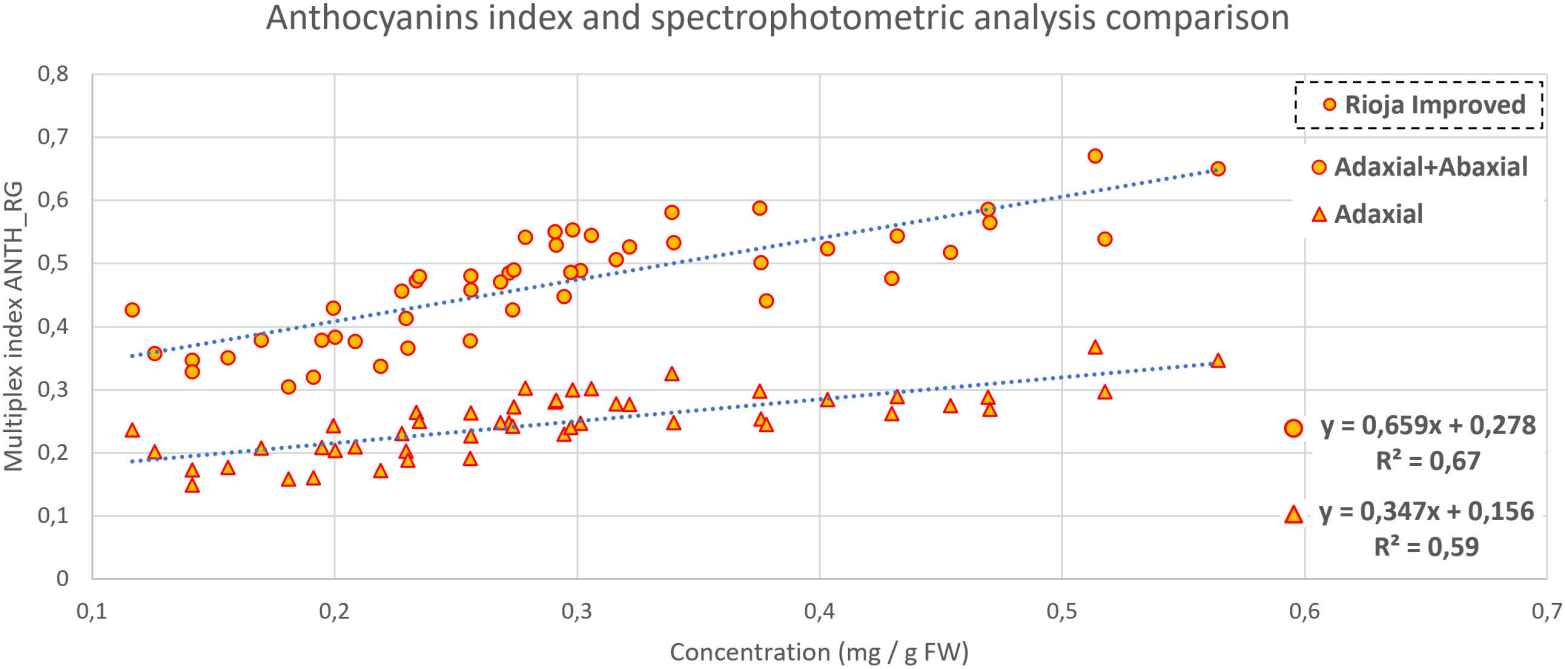
Anthocyanins content comparison between Multiplex (adaxial and both sides of cotyledon) index and concentration in rioja improved radish, with a P-value<0.001 in both cases.

**Figure 8:**
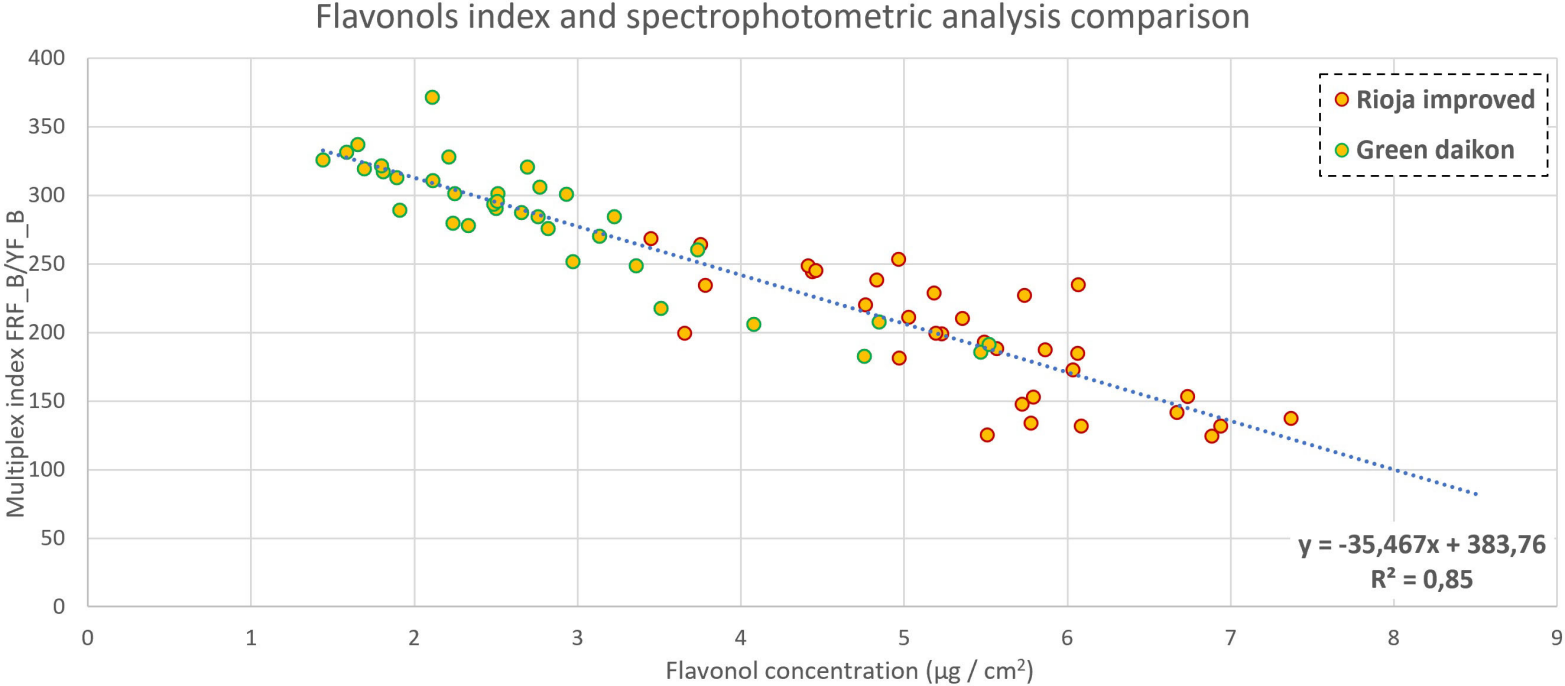
Flavonol content comparison between Multiplex index SFR (measuring adaxial side of cotyledon) and concentration in green daikon and rioja improved radish, with a P-value<0.001.

A comparison between Multiplex measures and spectrophotometric analyses is reported in **Figures 6**–**8**. The metabolite concentration from the spectrophotometric analysis was computed in the function of cotyledonal fresh weight (mg/gFW), area (mg/cm^2^), and both (mg/cm^2^gFW). Their correlation with Multiplex indexes was analyzed by computing the R^2^ parameter to evaluate the goodness of fit.

#### 3.3.1 Chlorophyll content

Multiplex automatically computes two indexes that are related to chlorophyll content: SFR_R_ and SFR_G_ (Buschmann, 2007). They did not show any significant correlation with chlorophyll accumulation measured with the spectrophotometric analysis, both for green and red radish microgreen cultivation. Multiplex measures 12 fluorescence indexes, and it is possible to compute 66 ratios between them. Each ratio was then compared with the extraction concentration measurements obtained to find an eventual good fit. For green daikon radish, the ratio FRF_B_/FRF_UV_ gave discrete results when correlated with total chlorophyll concentration, as reported in **Figure 6**. The R^2^ coefficient was 0.73, with a P-value<0.001. This index evaluates chlorophyll fluorescence in far-red regions, when excited with blue (Israsena Na Ayudhya et al., 2015) and UV light (Meyer et al., 2003). Fluorescence is typically measured in the far-red region, above 715 nm to avoid the red fluorescence re-absorption by chlorophyll when excited in the UV band (Meyer et al., 2003).

For red radish, the indexes showed no significant correlation with the data measured. This could be explained by the higher heterogeneity in red radish plants.

#### 3.3.2 Anthocyanin content

In **Figure 7**, two correlations (adaxial + abaxial readings and adaxial alone) between red radish anthocyanin concentration and ANTH_RG_ Multiplex index are reported. The ANTH_RG_ index is automatically computed during the measure of each sample, and its correlation with leaves’ and fruits’ anthocyanin content has been already demonstrated (Pfündel et al., 2007; Merzlyak et al., 2008). A correlation with R^2^ equal to 0.59 was found for the index measured on the cotyledons’ adaxial side, and, considering the sum of adaxial and abaxial, the R^2^ increased to 0.67.

#### 3.3.3 Flavonol content

Multiplex computes automatically the FLAV index, which has been demonstrated to be linked to flavonol content (Ounis et al., 2001; Bilger et al., 1997; Bilger, et al., 2001; Cerovic et al., 2002). In this experiment, both for red and green radish, FLAV did not show any significant correlation with the measured concentration. Among all the ratios between Multiplex indexes, the ratio FRF_B_/YF_B_ gave interesting results for both red and green radish. As shown in **Figure 8**, the correlation between this ratio and the concentration in the function of the cotyledonal area gave an R^2^ of 0.85, considering all the samples for both green and red radish. The index FRF_B_ is an indicator of chlorophyll fluorescence in the far-red region when excited with blue light (Israsena Na Ayudhya et al., 2015). Flavonols almost exclusively contribute to the fluorescence of the adaxial epidermal layer of leaves when excited with blue light, as discussed in Tattini et al. (2004). This phenomenon was observed by measuring the YF_B_, the yellow fluorescence under blue excitation. Therefore, the correlation with the flavonols’ measured content was obtained by scaling the flavonols’ fluorescence index (YF_B_) with the chlorophyll fluorescence index (FRF_B_).

## 4 Discussion

Our work aimed to observe the effects of different LED light recipes (with different percentages of red, blue, and green light), on the growth and secondary metabolite accumulation of two different cultivars of hydroponically grown radish microgreens (*green daikon* and r*ioja improved*, with high content of anthocyanins). In the first experimental trial, the L2 light (40% red, 20% green, 40% blue) effect on the morphological parameters was statistically significant: almost all the parameters (fresh and dry weight, hypocotyl length, and cotyledon weight and area) showed an increase under L2 light, reaching their maximum accumulation, while their minimum accumulation was observed under L1 light (20% red, 20% green, 60% blue). This reduction of biomass accumulation in plants grown under blue light has been already well described (Son and Oh, 2013; Vaštakaite et al., 2015; Brazaitytė et al., 2021). In red mustard microgreens, Brazaitytė et al. (2021) observed a reduction in biomass under a high percentage of blue light. However, in the same study, the highest yield was obtained with the same internal red-to-blue ratio of L3, while the 1:1 ratio (similar to L2) gave lower results in biomass accumulation (Brazaitytė et al., 2021). In Bantis (2021), it was demonstrated that a lower red-to-blue ratio light induced lower biomass accumulation in red mustard microgreens, while the different light treatments showed no effects on radish fresh and dry weight. Similar effects were described in Gerovac et al. (2016), where mustard microgreens grown under red/green/blue (74/18/8%) compared to red/blue (87/13%) and red/blue/far-red (84/9/7%) lights resulted in higher fresh weights. Similar results were obtained on many microgreens species (Gerovac et al., 2016; Bantis, 2021). In addition, Kong and Zheng (2020) observed no difference in fresh and dry weight on arugula, cabbage, kale, and red mustard microgreens under different light treatments. Our studies show that the effects of light on microgreen growth indicate a very complex interaction, as confirmed in the literature (Kyriacou et al., 2016).

The main objective of the first trial was to select the best light recipe to increase biomass accumulation in radish microgreens. The main parameter evaluated in this study is the dry weight, which is considered in the literature to be the best indicator of plant growth (Huang et al., 2019) due to its independence from plant irrigation status and health (Huang et al., 2019). The L2 light recipe induced the highest dry weight values, exhibiting a percent increase of +23% for green daikon radish and +39% for rioja improved radish compared to the least effective recipe. These results highlighted an efficient recipe to increase biomass while minimizing power consumption. Particularly, it is valuable in the context of long-term space missions where power consumption remains one of the most critical parameters.

Once the best light recipe, in terms of biomass accumulation, was found, far-red and UV were added, both together and separately, to the L2 recipe, and the effects on both growth and accumulation of secondary metabolites were observed. During this second trial, different results were obtained for green (**Table 3**) and red (**Table 4**) radishes due to cultivar-specific effects on the activation of the anthocyanin metabolic pathway, in the red variant reducing primary metabolism while in the green the opposite.

For green radish, the addition of UV and far-red alone induced a statistically significant reduction in all growth metrics except for hypocotyl length. UV and far-red light typically induce a specific response that alters the energy allocation in plants, moving the primary metabolism toward the production of secondary metabolite to prevent stress damage (Brazaityte et al., 2015; Brazaityte et al., 2019); He et al., 2021. This hypothesis is also confirmed by the higher flavonol concentration found in leaves of radish microgreens grown under UV and/or far-red supplemented LED lights. Compared to growth under the L2 treatment, hypocotyl lengths for green radish tended to be lower under UV and far-red lights alone; however, these heights were not significantly different. Meanwhile, the combined L2 + 10UV+10FR treatment resulted in significantly taller plants (hypocotyl elongation of +17%). It is well known in the literature that UV-A radiation can activate cryptochrome, a blue light receptor that inhibits plant elongation (Huché-Thélier et al., 2016). At the same time, despite sharing a common photoreceptor with blue light, UV-A appears to have a greater inhibitory effect on plant elongation than blue light (Kong et al., 2019b). Far-red light should have induced hypocotyl elongation because of the reduced phytochrome activity and triggered shade-avoidance responses (Franklin and Whitelam, 2005), as shown for some microgreens: arugula, mustard, cabbage, and kale (Ying et al., 2020). However, this response is very species-specific, and data for radish are still not present in the literature. The interaction between the two lights probably induced a different behavior in green radish microgreens.

Conversely, the reduced size of cotyledons (both in terms of area and weight), after the inclusion of far-red light, can be explained by the shade-avoidance response; in fact, in microgreens, one of the most common responses to shade is to reduce cotyledon size (Kong et al., 2019a). Since cotyledonal biomass in microgreens is about half of the total mass, a reduced total plant biomass was observed.

In this second trial, the concentrations of chlorophyll, anthocyanins, and flavonols were evaluated and compared between radish microgreens grown under different light recipes. In particular, it was observed that far-red light alone induced a moderate increase in total chlorophyll concentration, even though it was not in a statistically significant way. Similar results are reported in Gerovac et al. (2016), where Mizuna microgreens showed higher chlorophyll content when far-red radiation was added to the light recipe. However, in the same study, the opposite effect was observed in mustard and kohlrabi microgreens.

Conversely, the addition of UV and far-red, both alone and in combination, induced a higher accumulation of flavonol content with respect to the L2 recipe in a statistically significant way. In many studies, it has been reported that both far-red and UVA radiation stimulate secondary metabolite accumulation (Brazaityte et al., 2015; Brazaityte et al., 2019; He et al., 2021).

Rioja improved cultivation showed different results compared to green daikon: no statistically significant effects were observed on dry weight and cotyledon fresh weight among different light treatments. This red variety responded differently from its green counterpart, mainly due to its higher content of anthocyanins, a polyphenolic compound with powerful antioxidant properties (Ku et al., 2020), which counteracted light-induced stress. Higher anthocyanin concentration reduced the stressful effect of the UV and far-red lights, mitigating the effect observed on biomass accumulation for green radish and acting as a protective agent (Chalker-Scott, 1999; Quina et al., 2009). The response to UV-B (more energetic than UV-A) radiation in green and red lettuce has already been studied, showing that the red genotype was more resistant to UV radiation (Assumpção et al., 2019). This phenomenon is related to the accumulation of health-promoting compounds such as anthocyanins, which are naturally higher in red plants. On the contrary, the combination of UV and far-red light induced a significant increment in fresh total biomass compared to the control L2, probably due to a higher hydration level, considering that no increment in dry weight was observed. Under the same lights, a longer hypocotyl with an increment of +31% with respect to control was observed, showing the same trend seen in green radish.

The addition of UV light alone induced a significant increase in chlorophyll content compared to the control L2. The same trend has been already described in red lettuce (Lee et al., 2022), where, after 3- and 6-day exposure to UV-A light before harvest, a significant increase in chlorophyll content was observed; similar results were detected in red kale (Jiang et al., 2022). The combination of UV and far-red light, instead, induced a significant increase in anthocyanin concentration compared to other lights and control L2. It is well known that far-red and UV-A lights stimulate anthocyanin production, as shown in some research studies (Carvalho and Folta, 2016; Li and Kubota, 2009; Brazaitytė et al., 2016; Brazaitytė et al., 2019; He et al., 2021), while the combined effect of both lights has to be studied on radish microgreens. Hooks et al. (2022) studied the effect of far-red and/or UV radiation on basil, kale, cabbage, and kohlrabi microgreens, and a variation in anthocyanin concentration was not observed even though experimental conditions were different from the data here presented: the control light was a white LED with 100 μmol m^-2^s^-1^ of PPFD, and UV and far-red light were added having a total flux still equal to 100 μmol m^-2^s^-1^, therefore reducing white intensity. The experiment presented here worked with the addition of UV and far-red light to the control L2, having a total flux higher with respect to L2 alone. On the contrary, the introduction of far-red and the sum of far-red and UV lights induced a significant decrease in flavonol concentration compared to L2 light. This phenomenon can be explained by considering the metabolic pathway of anthocyanins, where flavonols are an intermediate product in anthocyanins synthesis (Winkel-Shirley, 2001). UV and/or far-red light are well known for inducing accumulation of anthocyanins (Carvalho and Folta, 2016; Li and Kubota, 2009; Brazaitytė et al., 2016; Brazaitytė et al., 2019; He et al., 2021), therefore an increment in the final bio-product (anthocyanins) corresponds to the reduction of the intermediate products (flavonols). A comparison between the non-destructive measures and spectrophotometric analyses is also reported to evaluate the degree of association between the two methods via a correlation analysis.

Multiplex automatically computes two indexes that are related to chlorophyll content: SFR_R and SFR_G (Buschmann, 2007). Their correlation in the case of microgreen cotyledon analysis has not yet been studied. However, SFR_R and SFR_G indexes observed both in green and red radish cultivation did not show any significant correlation with the concentration measured with the spectrophotometric analysis.

Multiplex measures 12 fluorescence indexes, and it is possible to compute 66 ratios between them. Each ratio was then compared with the extraction concentration measurements to find an eventual good fit. For green daikon radish, the ratio F_RFB/F_RFUV gave discrete results when correlated with total chlorophyll concentration, as reported in **Figure 6**. The R^2^ coefficient was 0.73, with a P-value<0.001. This index evaluates chlorophyll fluorescence in the far-red region when excited with blue (Israsena Na Ayudhya et al., 2015) and UV light (Meyer et al., 2003). Fluorescence is typically measured in the far-red region, above 715 nm to avoid the red fluorescence re-absorption by chlorophyll when excited in the UV band (Meyer et al., 2003). For red radish, the indexes showed no significant correlation with the data measured. This could be explained by the high heterogeneity in red radish plants, and each of the seedlings showed different color expressions (linked to metabolite content).

In **Figure 7**, two correlations (adaxial + abaxial readings and adaxial alone) between red radish anthocyanin concentration and ANTH_RG Multiplex index are reported. The ANTH_RG is automatically computed during the measure for each sample, and its correlation with leaves’ and fruits’ anthocyanin content has been already demonstrated (Pfündel et al., 2007; Merzlyak, et al., 2008). A correlation with R^2^ equal to 0.59 was found for the index measured on the cotyledons’ adaxial side, and, considering the sum of adaxial and abaxial, the R^2^ increased to 0.67. Probably these discrete results are due to the high heterogeneity present in red radish plants as observed with chlorophyll.

Multiplex automatically computes the FLAV index, which has been demonstrated to be linked to flavonol content (Bilger et al., 1997; Bilger et al., 2001; Ounis et al., 2001; Cerovic et al., 2002). However, its correlation with microgreen cotyledons has not yet been studied. In this experiment, both for red and green radish, the FLAV index did not show any significant correlation with the measured concentration. Among all the ratios between Multiplex indexes, the ratio FRF_B/Y_FB (infrared fluorescence excited with blue/yellow fluorescence excited with blue) gave interesting results for both red and green radish. As shown in **Figure 8**, the correlation between this ratio and the concentration of flavonol, in relation to the cotyledonal area, gave an R^2^ of 0.85, considering all the samples for both green and red radish. The index FRF_B is an indicator of chlorophyll fluorescence in the far-red region when excited with blue light (Israsena Na Ayudhya et al., 2015). Flavonol, almost exclusively, contributes to the fluorescence of the adaxial epidermal layer of leaves when excited with blue light, as discussed in Tattini et al. (2004). This phenomenon was observed by measuring the YF_B, yellow fluorescence under blue excitation. Therefore, the correlation with the flavonol measured content was obtained by scaling the flavonol fluorescence index (Y_FB) with the chlorophyll fluorescence index (FRF_B).

## 5 Conclusion

The results obtained in this work are important for future space agriculture applications since a non-destructive fluorescence technique made it possible to monitor the accumulation of plant metabolite and to find the best harvest time in hydroponically grown microgreens. The possibility to calibrate, in case of good correlations, Multiplex absolute readings of chlorophyll, flavonol, and anthocyanin indexes with spectrophotometric measures allows us to derive in similar test conditions (same genotype, same environmental conditions, same nutrient solution, etc.) total chlorophyll, flavonol, and anthocyanin concentrations. It is important to design and develop plant-based BLSS integrated with non-invasive imaging and fluorescence sensing technologies to obtain better yields and quality in ready-to-eat fresh foods. To support long-duration human space missions, monitoring plant health non-destructively through imaging also allows astronauts to detect plant stress early enough to make timely corrections before the onset of symptoms, preventing costly events such as crop loss.

## Data availability statement

The original contributions presented in the study are included in the article/supplementary material. Further inquiries can be directed to the corresponding authors.

## Author contributions

MG: Conceptualization, Data curation, Investigation, Methodology, Software, Validation, Writing – original draft, Writing – review & editing. CS: Data curation, Methodology, Writing – review & editing. CP: Writing – review & editing. FF: Writing – review & editing. EB: Methodology, Writing – review & editing. AD: Writing – review & editing. LN: Conceptualization, Funding acquisition, Investigation, Software, Supervision, Validation, Writing – original draft, Writing – review & editing. MV: Conceptualization, Data curation, Investigation, Methodology, Supervision, Writing – original draft, Writing – review & editing.
